# Mechanism-Driven Strategies for Reducing Fall Risk in the Elderly: A Multidisciplinary Review of Exercise Interventions

**DOI:** 10.3390/healthcare12232394

**Published:** 2024-11-29

**Authors:** Yuan-Ji Zhong, Qing Meng, Chun-Hsien Su

**Affiliations:** 1 School of Physical Education and Arts, Jiangxi University of Science and Technology, Ganzhou 341000, China; zhongyuanji@jxust.edu.cn; 2 School of Physical Education, Huaqiao University, Xiamen 361021, China; mq@hqu.edu.cn; 3 Sport and Health Research Center, Huaqiao University, Xiamen 361021, China; 4 Department of Exercise and Health Promotion, Chinese Culture University, Taipei City 111369, Taiwan

**Keywords:** fall prevention, elderly exercise interventions, balance and stability, muscle strengthening, cognitive and psychological benefits

## Abstract

Falls among older adults present a major public health challenge, causing significant physical, psychological, and economic consequences. Exercise interventions are a proven strategy to reduce fall risk by targeting biomechanical, physiological, and psychological factors. This review examines evidence from 155 studies published between 2004 and 2024, including systematic reviews, meta-analyses, randomized controlled trials, and cohort studies. Data were rigorously screened and extracted using predefined criteria, with studies sourced from PubMed, MEDLINE, EBSCO (EDS), and additional gray literature identified via Google Scholar. Key findings show that balance and strength training improves postural control, gait stability, and neuromuscular coordination, while resistance training mitigates sarcopenia and enhances joint mobility. Cognitive exercises enhance attention, spatial awareness, decision-making, and psychological benefits like reduced fear of falling and greater social engagement. Multidisciplinary approaches integrating physical, cognitive, and social components deliver the most significant impact. This review underscores the value of evidence-based exercise programs in promoting active aging and enhancing the quality of life for older adults.

## 1. Introduction

### 1.1. Background on Falls and Aging

Falls among older adults represent a primary public health concern, causing significant physical, psychological, and economic challenges. Each year, approximately 20 to 40 percent of older adults experience falls, often leading to fractures, traumatic brain injuries, and increased mortality rates [1]. Beyond physical harm, the psychological consequences, such as the fear of falling, frequently result in reduced physical activity, social isolation, and a diminished quality of life [1,2].

Several factors contribute to the risk of falls, including chronic health conditions, medication usage, and balance impairments. Cardiovascular issues further elevate these risks [3,4]. Gender differences also influence fall risk, with men and women facing distinct challenges related to medication use and nutritional status [5].

Exercise interventions, particularly balance and resistance training, have effectively improved physical performance and reduced the likelihood of falls [1,4]. Programs like the falling safely training (FAST) illustrate the importance of adopting multifaceted strategies [6]. However, the persistently high incidence of falls highlights the need for comprehensive approaches that address the complex interplay of these risk factors [3,4].

In hospital settings, fall prevention remains a critical issue. Hospitalized older adults are especially vulnerable due to factors such as reduced mobility, acute medical conditions, and the effects of medications. Recent research from Italy has identified significant gaps in clinical risk management and fall prevention protocols in healthcare environments, underscoring the necessity for multidisciplinary interventions tailored to hospital settings [7].

As the global population continues to age, prioritizing fall prevention is crucial. Exercise-based interventions, which address physical, cognitive, and psychological dimensions, have shown promise in strengthening muscles, enhancing postural stability, and improving cognitive abilities such as attention and spatial awareness. A deeper understanding of these mechanisms can support the development of targeted programs that enhance the quality of life and promote independence for older adults, thereby fostering holistic health outcomes.

### 1.2. Rationale for Physical Activity or Exercise Interventions

Regular physical activity is crucial for mitigating age-related declines such as muscle and strength loss, reduced joint flexibility, impaired balance, and diminished neuromuscular coordination, all heightening fall risk in older adults. Evidence supports that regular physical activity enhances motor skills, mental health, and cardiovascular function, thereby promoting mobility and independence [8]. Resistance training combats sarcopenia, while balance exercises improve postural stability, which is essential for fall prevention [8,9].

A physically active lifestyle enhances static and dynamic balance, which is critical for postural control and the reduction in fall risk [10]. Tailored interventions significantly improve physical fitness and functional outcomes, as seen in studies involving older adults, including those with disabilities [9,11]. Poor physical performance correlates with higher fall risk and mortality, emphasizing the importance of regular strength and balance training to support mobility and cognitive functions [8,12].

Exercise interventions also enhance mental and emotional well-being. Fear of falling, a common psychological barrier, can reduce activity and accelerate physical decline. Exercise programs build confidence and improve mental health, supporting sustained activity and reducing fall risk.

Comprehensive exercise interventions address physical strength, balance, and psychological factors, mitigating fear and promoting well-being. This holistic approach empowers older adults, fostering independence and improving their quality of life.

### 1.3. The Objective of This Review

This review aims to synthesize evidence on the effectiveness of exercise interventions in reducing fall risk among older adults by exploring the underlying biomechanical, physiological, and psychological mechanisms. Guided by the PICO framework, the population includes older adults, both community-dwelling and at-risk individuals; the intervention focuses on exercise-based strategies such as balance training, strength training, and cognitive exercises; the comparison involves no intervention, minimal intervention, or alternative fall prevention methods; and the outcomes assessed include improvements in balance, stability, gait mechanics, muscle strength, cognitive function, and psychological well-being, leading to reduced fall incidence and enhanced quality of life. The review seeks to inform evidence-based practices for developing targeted, multidisciplinary exercise programs to address fall prevention in the elderly comprehensively.

## 2. Methodology

A comprehensive search was conducted to retrieve the relevant literature on the effectiveness of exercise interventions in reducing fall risk among older adults. This process included the following steps:

### 2.1. Databases and Search Resources

The search targeted multiple electronic databases to ensure a wide range of high-quality sources. The databases searched were PubMed, MEDLINE, and EBSCO (EDS). In addition to databases, the search also utilized Google Scholar as a supplementary tool to identify gray literature and additional studies not indexed in traditional databases. Google Scholar was specifically used to access conference proceedings, unpublished works, and institutional reports that might be overlooked. It was not considered a database but served as a resource for broadening the search scope.

### 2.2. Search Strategy

**Figure 1:**
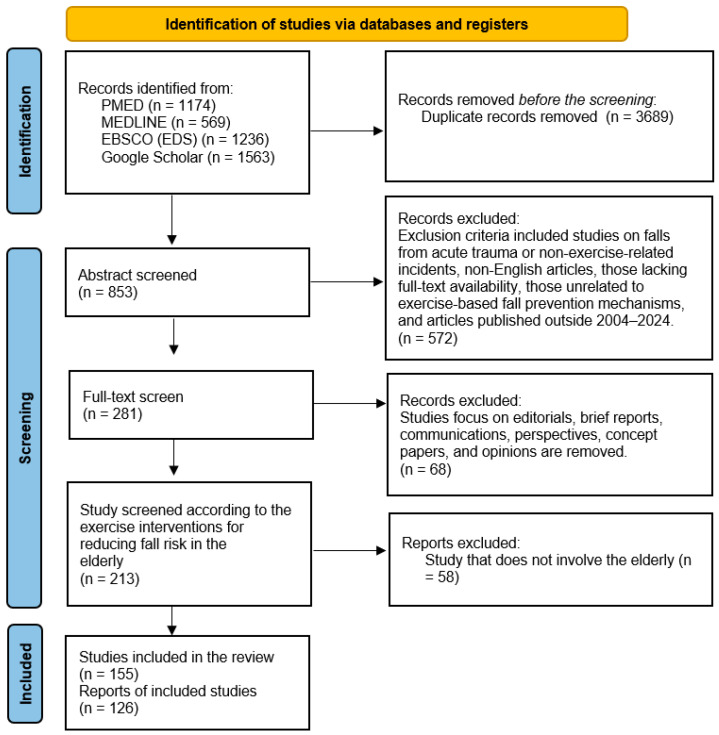
Flow diagram for the selection of original articles.

A standardized search protocol was implemented using predefined keywords such as “fall prevention”, “elderly”, “biomechanics”, “physiology”, “psychological impacts”, “exercise intervention”, “physical activity”, and “risk assessment”. Boolean operators (AND, OR, and NOT) were applied to refine the search and improve precision. For instance, queries like (“fall prevention” AND “elderly”) or (“exercise intervention” AND “biomechanics”) were utilized across databases to identify relevant studies. The detailed search strategy is presented in Figure 1 to enhance transparency and credibility.

### 2.3. The Chronological Flow of the Search Process

The search process followed a systematic chronological flow to ensure comprehensive coverage of relevant studies. Initially, searches were conducted in PubMed, MEDLINE, and EBSCO (EDS) using standardized keywords and Boolean operators. This was followed by a search in Google Scholar to identify gray literature, focusing on conference papers and institutional reports. References from critical articles were then reviewed to identify additional relevant studies. Finally, data were extracted from the identified studies, and each was screened for inclusion based on the predefined criteria.

### 2.4. Inclusion and Exclusion Criteria

The inclusion criteria for this review were designed to ensure a focused and relevant synthesis of evidence. Studies were included if they involved participants aged 60 or older, representing the population most at risk of falls and related complications. Articles published between 2004 and 2024 were considered to capture contemporary research and advancements in exercise interventions and fall prevention strategies. The review included studies involving older adults with various conditions to reflect real-world applicability. Still, it excluded those focusing solely on falls caused by acute trauma or non-exercise-related incidents, such as accidents unrelated to physical health. The mechanisms for fall prevention strategies were defined as biomechanical, physiological, or psychological processes improved through exercise interventions, such as enhanced balance, joint stability, muscle strength, or reduced fear of falling. Eligible studies included full-text articles written in English, encompassing qualitative and quantitative designs such as systematic reviews, meta-analyses, randomized controlled trials, cohort studies, and expert opinions.

### 2.5. Thematic Categorization

The reviewed studies were thematically categorized into the following three key mechanisms: biomechanical, physiological, and psychological. The biomechanical category encompassed studies focusing on balance, gait, posture, joint stability, and proprioception, with interventions such as resistance training, core exercises, and dynamic stability exercises. These studies reported outcomes including improved postural control, joint flexibility, and resilience against falls. The physiological category emphasized the biological and functional changes driven by exercise, including muscle hypertrophy, neuromuscular adaptations, and bone density improvements. Interventions like resistance and weight-bearing exercises demonstrated reduced sarcopenia, enhanced reaction times, and increased functional strength. Lastly, the psychological category addressed fear reduction, confidence building, cognitive enhancement, and mood improvement through dual-task training, mindfulness-based exercises, and group activities. Outcomes in this domain included reduced fear of falling, improved attention, and enhanced mental well-being, reflecting the multifaceted benefits of exercise in fall prevention.

## 3. The Biomechanical Mechanisms of Fall Prevention Through Exercise

### 3.1. Balance and Postural Control

Exercise interventions are vital for improving the biomechanical functions necessary for fall prevention in older adults by enhancing balance, postural control, and proprioception. These abilities are critical for maintaining stability and preventing falls. Research shows that balance and functional exercises can reduce fall rates by 23% and decrease the number of individuals who experience falls by 15%. Various exercise modalities, including balance, functional, and resistance training, have proven effective in mitigating fall risk, with Tai Chi emerging as a particularly effective intervention [13,14].

Proprioception, the body’s ability to sense its position and movement, is significantly improved through balance-focused activities such as single-leg stances and balance board exercises. These activities activate proprioceptors in muscles, tendons, and joints, improving sensitivity and responsiveness. Regular physical activity helps preserve proprioception in older adults, with physically active individuals demonstrating superior proprioceptive function compared to their sedentary counterparts [15,16]. Enhanced proprioception supports more precise postural adjustments, improving balance and reducing the risk of falls.

Balance exercises also require the coordinated activation of multiple muscle groups facilitated by the neuromuscular system, which includes the brain, spinal cord, and neural networks. A systematic review of 20 studies highlighted the significant positive correlations between trunk muscle strength, functional performance, and fall prevention. Core strength training and Pilates were identified as effective and feasible programs, showing high adherence rates and improving strength, balance, functional performance, and reduced fall risk among older adults. The repeated practice of these exercises enhances the timing and sequencing of muscle contractions, fostering efficient movement patterns and quicker reflexive responses to disturbances, which enable timely corrective actions to prevent falls [17,18,19,20].

Strength training targeting the core and lower limb muscles is essential for maintaining upright posture and stability. Research has shown that progressive resistance training improves physical capacity, gait speed, and muscle strength in older adults. These exercises promote muscle hypertrophy and strength, enhancing joint support and enabling effective corrective movements during physical activity [21,22].

Dynamic stretching and functional exercises further contribute to fall prevention by improving joint stability and flexibility. These activities increase the range of motion and enhance shock absorption, which is critical for safe mobility in diverse environments. Core training, which targets local and global spinal stabilization muscles, has improved trunk flexor and extensor strength. For example, Swiss-ball core training has demonstrated significant benefits in spinal stability and overall muscle strength [23,24,25].

Additionally, exercises incorporating visual, vestibular, and proprioceptive inputs—such as navigating obstacle courses or performing balance training on unstable surfaces—improve the brain’s ability to process sensory information. This capability is crucial for adapting to dynamic conditions and maintaining balance [19,26]. While balance and postural control are central to fall prevention, their effectiveness is significantly enhanced with exercises targeting gait and lower limb strength, promoting safe and efficient movement.

### 3.2. Gait and Lower Limb Strength

Enhancing gait mechanics and lower limb strength is a fundamental biomechanical strategy for fall prevention, particularly for older adults who often experience muscle atrophy and decreased stability [27,28]. Proper gait relies on the coordinated action of muscles, bones, and joints to ensure stable and efficient movement. Research highlights that exercise interventions, including resistance, power, coordination, and multimodal programs, can mitigate age-related gait speed and mobility declines. These programs improve balance, refine movement precision, and enhance the ability to absorb impact forces, ultimately reducing fall risk [29].

Lower limb strength is critical for essential movements such as walking, climbing stairs, and transitioning between sitting and standing [30,31]. Age-related sarcopenia, characterized by reduced muscle mass and fiber size, diminishes force production and adversely impacts gait [28]. Resistance training exercises, such as squats, leg presses, and lunges, effectively counteract these declines by promoting muscle hypertrophy and neuromuscular activation, improving muscle size and function. Evidence suggests that resistance training, whether performed independently or as part of a multimodal approach, significantly enhances muscle strength, power, mass, and functional capacity while reducing fall risk in frail older adults. Recommended training protocols involve 1–6 weekly sessions, with 1–3 sets of 6–15 repetitions at an intensity of 30–70% of one-repetition maximum (1-RM) for optimal outcomes [32,33]. Strengthened muscles provide better joint support and enable more controlled limb movements, essential for maintaining balance.

Gait performance depends on muscle strength, joint mobility, and neuromuscular coordination [34]. Weak lower limbs can result in compensatory gait patterns, such as shorter stride lengths and slower walking speeds, which increase fall risk [35]. Strength training improves muscle power and endurance, helping to maintain normal gait patterns and enhance shock absorption during movement, thereby reducing joint stress and minimizing the risk of injury [36]. Resistance training also refines neuromuscular coordination by improving motor unit synchronization and firing rates, resulting in smoother and more controlled movements [37]. These improvements are essential for navigating varied terrains and responding to unexpected obstacles, allowing for rapid corrective actions to maintain balance [38].

Walking exercises on uneven terrains provide additional challenges to the gait system, promoting adaptations that enhance dynamic stability. Studies show that increased terrain unevenness leads to greater variability in step duration, sacral excursion, and perceived stability. However, such exercises improve postural control metrics, including standing time on wobble boards and balance mats, anterior–posterior displacement, and power spectrum measurements [39,40]. These activities activate the proprioceptive and vestibular systems, improving responsiveness to environmental changes and reinforcing gait stability [41].

Physical exercise interventions have been shown to reduce fall rates and risk in healthy older adults significantly. Post-exercise stability and perturbation training further enhance the retention of fall prevention benefits by bolstering cardiovascular and muscular endurance, enabling sustained physical activity without undue fatigue [42]. Strengthening gait mechanics and lower limb function creates a robust foundation for joint stability, essential for maintaining overall balance and preventing falls [43,44].

### 3.3. Joint Stability and Range of Motion

Maintaining stability and an optimal range of motion in critical joints, such as the knees, hips, and ankles, is essential for postural control, efficient movement, and fall prevention through exercise [45,46]. Flexibility exercises, such as gentle stretching, play a key role in reducing joint stiffness and enhancing the extensibility of muscles and connective tissues, which improves joint mobility and mitigates the risk of falls. Evidence suggests that combining strength training with dynamic and static stretching in exercise programs significantly improves flexibility in older adults, reduces fall risk, and enhances the overall quality of life [47]. Specific exercises targeting hip flexibility, such as hip circles or seated hip openers, have increased the hip range of motion, reduced muscle tightness, and improved joint capsule elasticity, facilitating smoother transitions during movement and greater stability [48].

Knee-focused exercises, including hamstring and calf stretches, improve knee mobility by enhancing the functional length of muscles, thereby reducing strain during activities such as bending or weight shifting [49]. Similarly, ankle mobility exercises, such as ankle rotations and dorsiflexion movements, are critical for improving ankle stability and flexibility, essential for maintaining balance and adapting gait to various surfaces. Studies have demonstrated that combining joint mobilization with targeted training significantly enhances ankle dorsiflexion mobility, reduces self-reported ankle instability, and improves balance performance, particularly in the posterolateral direction, compared to training alone or no intervention [50,51]. Considering the ankle’s pivotal role in controlling the body’s center of gravity, reduced flexibility or strength in this joint can impair postural adjustments and restrict movement, particularly on uneven surfaces or inclines [52].

Enhancing joint range of motion and functionality through flexibility and mobility exercises also improves the sensitivity of mechanoreceptors within the joints, leading to superior proprioceptive feedback and better postural control [53]. Flexible joints are more effective at absorbing impact forces and redistributing loads, thereby minimizing the risk of injury [54]. Research indicates that older adults who regularly engage in flexibility training demonstrate improved joint stability, enhanced shock absorption, and a lower likelihood of fall-related injuries due to their ability to recover quickly from sudden movements [47,53]. Joint stability and flexibility provide the structural support necessary for dynamic stability, allowing the body to maintain balance during complex or unexpected movements. This structural integrity directly contributes to a reduced risk of falls and improves overall functional performance.

### 3.4. Dynamic Stability

Dynamic stability is a critical aspect of fall prevention in older adults, involving maintaining balance and control during movement while effectively responding to sudden shifts or disturbances [55,56]. This capability integrates sensory inputs, neuromuscular responses, and musculoskeletal functions to adapt to environmental changes and maintain stability [57]. Exercise interventions targeting dynamic stability, such as step-up routines, agility drills, and lateral movements, enhance neuromuscular coordination and improve proprioception. Evidence indicates that dynamic exercises incorporating proprioceptive neuromuscular facilitation patterns can enhance posture and trunk stability [58].

Step-up exercises simulate everyday actions such as stair climbing and navigating uneven surfaces. These movements train the lower limbs and core muscles to handle vertical displacements while maintaining balance. Resistance and strength training programs aimed at improving muscle strength and coordination have been shown to effectively reduce fall risk in older adults by enhancing leg–core integration, which provides a stable foundation for dynamic movements [59,60]. Similarly, agility drills, such as side steps, cone weaves, and shuttle runs, are essential for improving lateral stability by training the body to control side-to-side motion. This is particularly significant because many falls occur due to lateral loss of balance. Agility training also sharpens reflexes and reduces reaction times, enabling faster corrections during balance disturbances [61,62].

Research has reviewed various physical exercise programs to improve static and dynamic balance in older adults. Interventions including resistance and aerobic training, balance-focused exercises, and specialized tools like T-Bow^©^ and wobble boards consistently demonstrate improvements in balance measures. Combined exercise programs have shown the most significant impact, optimizing neuromuscular control by refining the timing and sequencing of muscle activations essential for dynamic balance [17,54]. Enhanced proprioceptive feedback from these activities further improves joint positioning and movement detection, both critical for maintaining stability during active motion [63].

Dynamic stability exercises offer a comprehensive approach to balance enhancement and fall prevention by significantly improving mobility and reducing fall risk. They achieve this by targeting sensory–motor integration, essential for effective balance control [64]. These exercises provide a holistic solution to maintaining dynamic stability in older adults, addressing the underlying biomechanical and neuromuscular mechanisms. Table 1 in this review summarizes the biomechanical mechanisms of fall prevention through exercise.

## 4. Physiological Mechanisms Underlying Fall Prevention

### 4.1. Muscle Hypertrophy and Sarcopenia Mitigation

Sarcopenia, the age-related decline in muscle mass and strength, is a significant factor contributing to the increased risk of falls in older adults [28,31]. This condition results from reduced muscle protein synthesis, hormonal changes—such as lower levels of anabolic hormones like growth hormone and testosterone—and decreased physical activity, reducing both the size and number of muscle fibers [65]. Resistance training has been shown to effectively combat these effects by promoting muscle hypertrophy, increasing muscle cross-sectional area and strength, and enhancing overall functional capacity. Studies confirm that resistance training, whether implemented independently or as part of a multimodal program, significantly improves maximal strength, muscle mass, muscle power, and functional independence, reducing fall risk in older adults. Optimal outcomes are achieved through carefully designed training protocols that specify frequency, volume, and intensity [32,66].

Resistance exercises, such as squats, leg presses, and arm curls, target key muscle groups in the legs, core, and arms, stimulating muscle protein synthesis, activating satellite cells, and facilitating muscle fiber recruitment [30]. Regular resistance training has been shown to particularly benefit type II fast-twitch muscle fibers, which are crucial for generating the rapid and powerful movements necessary to prevent falls. Age-related muscle decline is largely attributed to atrophy in type II fibers; therefore, resistance training’s ability to restore and enhance these fibers directly combats the loss of muscle mass and strength associated with aging [67,68]. This improvement provides better physical support and stability, enhances functional independence, and reduces reliance on external assistance.

Aging is also associated with the progressive loss of spinal motor neurons due to apoptosis, reduced insulin-like growth factor I (IGF-1) signaling, increased circulating pro-inflammatory cytokines (e.g., TNF-α, TNF-β, and IL-6), and heightened oxidative stress. Resistance training mitigates these effects by enhancing neuromuscular function and improving motor unit recruitment and synchronization, which leads to faster reaction times and better balance [37]. Additionally, resistance training stimulates the release of anabolic hormones, such as growth hormone and IGF-1, which support muscle repair and growth [69]. By addressing sarcopenia and enhancing muscle tissue strength, resistance training improves physical capacity and contributes to safer and more controlled movements, thereby playing a critical role in fall prevention [8,69,70].

### 4.2. Neuromuscular Adaptations

Exercise is critical in improving neuromuscular coordination, an essential factor in fall prevention for older adults [36,71]. Strength and balance exercises enhance motor unit recruitment and synchronization, increasing the number and efficiency of motor units activated during movement. A motor unit, composed of a motor neuron and the muscle fibers it controls, is the fundamental functional unit of muscle contraction. Research has demonstrated that four weeks of strength training with isometric voluntary contractions can significantly alter motor unit discharge characteristics in the tibialis anterior muscle. These adaptations include increased motor unit discharge rates, reduced recruitment-threshold force of motor units, and stable input–output gain of motor neurons [72]. Improved recruitment enables the body to generate the necessary force to maintain or regain balance after a stumble or slip [73]. Strength-training exercises like lunges and step-ups strengthen neural pathways between the brain and muscles, resulting in quicker and more precise muscle responses [74].

Balance-focused exercises, such as single-leg stands and stability ball activities, further enhance neuromuscular control by requiring continuous posture and muscle tension adjustments to maintain stability. Evidence from 20 randomized clinical trials indicates that balance training significantly improves postural sway and functional balance compared to untrained control groups, with greater improvements observed in longer training programs [18,75]. These exercises engage the central and peripheral nervous systems, enhancing proprioception and spatial awareness [76]. Improved proprioception allows for more accurate body position and movement detection, enabling rapid and effective adjustments when balance is disrupted [77].

Research has shown that older adults prone to falls often struggle to recruit appropriate motor modules for walking and balance control. This reduced motor repertoire limits their ability to respond effectively to balance challenges, increasing their fall risk [78]. Exercise interventions address this limitation by improving motor unit activation and neural efficiency, enabling older adults to handle balance disturbances better and maintain stability. Through these neuromuscular adaptations, exercise directly supports the physiological mechanisms necessary for stability and safer movement in daily life activities [79].

### 4.3. Bone Density and Joint Integrity

Age-related bone density loss, known as osteopenia or osteoporosis, significantly increases the risk of fractures from falls, particularly in older adults and postmenopausal women. Hormonal changes, such as reduced estrogen levels, disrupt bone metabolism, leading to an imbalance where bone resorption exceeds bone formation. This results in diminished mineral content and weakened bone structures [80,81,82]. Weight-bearing and resistance exercises are crucial in counteracting these effects by promoting bone remodeling. Mechanical stress applied to the skeletal system during these activities stimulates osteoblast activity, encouraging the formation of new bone tissue [83].

Exercises such as walking, stair climbing, and resistance training exert compressive and tensile forces on weight-bearing bones, particularly in the spine, hips, and legs. Research has shown that such loading exercises reduce fat mass, improve handgrip strength, enhance postural sway, and increase bone mineral density, especially at critical sites like the femoral neck. These improvements are accompanied by gains in various physical functions [84]. The mechanical stress generated during these activities facilitates calcium and mineral deposition, reinforcing cortical and trabecular bone structures. This reinforcement improves the bone’s ability to withstand impacts, reducing fracture risk during falls [85].

In addition to strengthening bones, exercise supports joint integrity by improving the strength of the muscles, tendons, and ligaments that stabilize joints. Enhanced muscle strength reduces stress on joint surfaces and promotes proper alignment, while stronger tendons and ligaments enhance joint stability and flexibility [86]. By maintaining bone density and joint health, weight-bearing and resistance exercises decrease fall risk and reduce the severity of injuries in the event of a fall. These benefits support mobility and help preserve the quality of life for older adults.

### 4.4. Cardiovascular and Endurance Benefits

Cardiovascular endurance is a vital physiological factor in fall prevention, as it influences an individual’s ability to remain active, responsive, and balanced over extended periods [87]. Aerobic exercises, such as brisk walking, cycling, and swimming, significantly enhance cardiovascular health by improving cardiac output, lung capacity, and overall stamina. Studies show that regular aerobic exercise over 24 weeks improves flow-mediated dilation and reduces pulse wave velocity in obese and overweight older adults, indicating better vascular function and reduced arterial stiffness [88]. These benefits result from physiological adaptations such as increased stroke volume, greater capillary density in muscle tissues, and enhanced mitochondrial efficiency, all of which contribute to more efficient oxygen utilization [88].

Aging often leads to a decline in cardiovascular function, causing increased fatigue and reduced endurance, adversely impacting balance and stability [85]. Fatigue can weaken muscle function and impair proprioceptive abilities, making physical tasks more challenging and increasing the risk of falls. For example, older adults may become fatigued more quickly during activities like cooking, cleaning, or gardening, which can delay reaction times and hinder their ability to correct balance disturbances effectively.

Improved cardiovascular endurance helps mitigate these risks by reducing fatigue and enabling older adults to perform daily activities more efficiently. A meta-analysis found that moderate-intensity aerobic sessions lasting 45–60 min are associated with cognitive benefits, including enhanced attention and reaction times [89]. Enhanced aerobic capacity supports better muscle oxygen and nutrient delivery, facilitating sustained physical function and improved postural control. Research further indicates that higher aerobic capacity correlates with better metabolic profiles in muscle tissue and white adipose tissue (WAT), enhancing energy metabolism and promoting greater endurance during physical activities [90].

Greater cardiovascular endurance improves balance during prolonged activities. It helps conserve energy for essential daily routines such as shopping, housework, and social interactions, thereby supporting independence and overall quality of life [91]. Additionally, aerobic exercise enhances cognitive functions like attention and decision-making, which are crucial for environmental awareness and quick reactions to prevent falls [90]. Regular exercise directly supports the physiological mechanisms essential for fall prevention by improving cardiovascular endurance, enabling sustained physical activity, and reducing the risks associated with fatigue and coordination deficits [91]. For more information on the physiological mechanisms underlying fall prevention, see Table 2.

## 5. Psychological Mechanisms in Exercise-Based Fall Prevention

### 5.1. Fear Reduction and Confidence Building

The fear of falling is a significant psychological barrier for older adults, often leading to reduced mobility, decreased physical activity, and a cycle of physical decline [92,93]. This fear limits participation in daily activities, increases social isolation, and diminishes quality of life [93]. Physiologically, anxiety related to falling can manifest as muscle tension and changes in gait and balance, such as slower walking speeds and increased postural sway, which paradoxically heighten the risk of falls. Psychological factors play a critical role in fall risk, highlighting the importance of assessing physiological and perceived risks to effectively tailor fall prevention interventions for older adults [94].

Exercise programs incorporating psychological elements, such as cognitive–behavioral approaches, have been shown to reduce fear and build confidence [95]. Techniques like positive reinforcement, goal setting, and gradual exposure to increasingly challenging movements allow older adults to confront and manage their fear systematically [96]. Initiating exercise with low-impact activities, such as seated movements or gentle walking, helps participants regain confidence in their physical capabilities before progressing to more complex exercises [97]. Mindfulness and relaxation techniques reduce anxiety and enhance focus during exercise sessions, contributing to a more positive and empowering experience [97].

Balance training and strength conditioning are particularly effective in alleviating fear by improving physical stability and body control [98]. The enhanced sense of control, reinforced through encouragement from instructors and peers, fosters self-efficacy and confidence [99]. Group exercise settings promote social interaction, boosting motivation and program adherence [99]. Over time, increased confidence encourages greater participation in physical activities, reducing social isolation and inactivity while lowering fall risk [100].

Addressing the psychological components of fall prevention is integral to comprehensive strategies targeting physical and mental health. Reducing fear and fostering confidence supports emotional well-being and improves cognitive functioning, as decreased anxiety enhances focus and decision-making during movement. These combined benefits are foundational for effective fall prevention interventions [95,100].

### 5.2. Cognitive Enhancement

Cognitive function is essential for safe movement, particularly in complex environments that require quick decision-making, spatial awareness, and focused attention to navigate obstacles effectively [101,102]. However, age-related cognitive decline can impair these abilities, increasing the risk of falls among older adults [103]. Empirical evidence demonstrates that exercise enhances key cognitive functions related to mobility and fall prevention, including attention, executive function, and spatial awareness [103,104]. Physical activity promotes neurogenesis and enhances synaptic plasticity, improving communication between brain cells. Studies show that aerobic exercise can prevent age-related hippocampal deterioration and support neuronal health, particularly in the left hemisphere [105]. Exercises such as walking, dancing, and Tai Chi activate multiple brain regions associated with memory, processing speed, and spatial perception. A meta-analysis of 29 studies involving 2049 participants found that aerobic training led to modest improvements in attention, processing speed, executive function, and memory [106].

Improved cognitive function translates to better environmental awareness, quicker decision-making, and enhanced attentiveness, all crucial for fall prevention [107]. Magnetic resonance imaging (MRI) studies have explored the relationships between physical activity, cognitive performance, and brain structure across age groups, differentiating between the effects of metabolic exercises (e.g., cardiovascular and resistance training) and coordinative exercises (e.g., balance and coordination training) on energy metabolism and cognitive processes [107,108]. Additionally, exercise has improved dual-task performance, which involves managing cognitive tasks while performing physical activities—a skill frequently required in daily life [109]. Exercise also supports brain health by increasing cerebral blood flow and promoting the release of neurotrophic factors, such as brain-derived neurotrophic factor (BDNF), which enhance neuronal health and longevity [109]. These cognitive benefits help older adults maintain mental sharpness, reducing the risk of falls caused by lapses in attention or judgment [97].

Combining physical exercise and cognitive training offers a synergistic effect on cognitive function. Research highlights that spatial perception, self-motion sensing, and variations in brain microstructure can influence an individual’s navigational performance [101]. Interventions that integrate physical and cognitive components have demonstrated greater improvements in executive function than those focusing on a single aspect [101]. By addressing both physical and cognitive dimensions, exercise programs comprehensively enhance the psychological mechanisms underlying fall prevention. Furthermore, improved cognitive function reinforces psychological benefits, as enhanced mental acuity boosts confidence and reduces anxiety, contributing to safer and more active lifestyles.

### 5.3. Mood and Motivation

Exercise has a well-documented positive impact on mood, largely due to the release of endorphins and neurotransmitters such as dopamine and serotonin, which are associated with enhanced well-being and reduced stress levels [110,111]. Improved mood through physical activity can alleviate symptoms of anxiety and depression—common conditions among older adults that negatively affect motivation and engagement in daily activities. A study involving older adults assigned to either a dual-task or resistance exercise program over six weeks found that both programs significantly improved cognitive function, mood, depression symptoms, functional fitness, and activities of daily living. Dual-task resistance exercise, in particular, demonstrated superior cognitive benefits for older adults with cognitive impairments [112]. By elevating mood, exercise increases the likelihood of sustained participation in physical activities, contributing to long-term fall prevention.

Tailored exercise programs for older adults, including aerobic training, strength exercises, flexibility routines, and balance training, have shown significant benefits for mental health. These interventions support psychological well-being by modulating the hypothalamus–pituitary–adrenal (HPA) axis and improving sleep quality. Research has also demonstrated that regular physical activity enhances cognitive function, reduces depressive symptoms, and fosters positive emotions in older adults [113].

In addition to its direct psychological benefits, exercise combats social isolation and loneliness, mainly through group-based activities such as aerobics or Tai Chi. Social interactions in these settings provide mutual support and encouragement, positively impacting mood and motivation [114,115]. A supportive social environment fosters adherence to exercise programs, crucial for achieving sustained improvements in balance and strength and reducing fall risk [116].

Moreover, the psychological benefits of exercise, including increased self-efficacy and a sense of accomplishment, motivate older adults to maintain an active lifestyle [117]. This creates a positive feedback loop, where improved mood and heightened motivation promote consistent engagement in physical activity, reinforcing sustainable behaviors for fall prevention [118]. By addressing mood and motivation within exercise interventions, psychological mechanisms are leveraged to enhance adherence and reduce fall risk in older adults. These improvements also facilitate greater social engagement, reinforcing a cycle of participation and commitment to regular physical activity [119].

### 5.4. Social Influence and Support

Social interaction is crucial in promoting physical activity, especially for older adults who may experience loneliness or social isolation [114,120]. Group exercise settings provide a supportive and engaging environment where participants can connect, share experiences, and receive encouragement. Research informed by self-categorization theory highlights the effectiveness of community-based, age-targeted exercise programs for older adults rather than gender-specific approaches [121]. Group activities, such as senior fitness classes and walking groups, foster a sense of belonging and accountability, which are critical for consistent participation. Key motivators for engaging in physical activity include perceived physical and mental health benefits, positive social influences, observing health deterioration in peers, and a desire to spend time with and support family members. Conversely, barriers include preexisting health conditions, fear of injury, negative social influences, lack of time and motivation, inconvenient scheduling, and financial costs [122].

The presence of peers in group exercise settings reduces psychological barriers often associated with solitary exercise. Participants benefit from encouragement, reassurance, and a collective sense of purpose, which enhances emotional well-being, reduces isolation, and increases motivation to stay active [123,124]. Instructors are essential in creating an inclusive and positive atmosphere by offering guidance, encouragement, and exercise modifications tailored to an individual’s capabilities. This support from instructors promotes adherence to exercise programs and enhances participants’ self-efficacy and confidence in their physical abilities [125].

Social support within group exercise programs also builds psychological resilience in older adults by providing emotional reinforcement and practical assistance, helping to alleviate anxiety and fear of injury [126]. The sense of community fostered in these programs creates a positive feedback loop, where increased participation leads to better physical and psychological health, encouraging sustained engagement in physical activity [127]. By leveraging social influence and support, exercise interventions effectively address the psychological mechanisms essential for fall prevention, enhancing mental well-being and physical stability. For further insights into the psychological mechanisms underpinning exercise-based fall prevention, see Table 3.

## 6. Integrated Mechanisms in Multidisciplinary Approaches

### 6.1. Coordination Between Physical and Cognitive Benefits

Preventing falls among older adults requires a comprehensive approach addressing physical and cognitive factors critical to stability, mobility, and decision-making [128]. Exercise interventions combining physical and cognitive training enhance body strength and cognitive function [129,130]. Activities such as Tai Chi, dance, and balance-focused exercises simultaneously engage muscular and cognitive systems. As participants perform structured movements, these exercises demand postural control, attentional focus, spatial awareness, and memory recall. A review of 20 studies involving 2553 participants, including randomized controlled trials (RCTs) and observational studies, found that Tai Chi significantly improved cognitive function, particularly executive functioning in cognitively healthy adults and global cognitive function in those with cognitive impairments [131].

Dual-task exercises improve neuromuscular communication, enhancing mental focus and reaction time. Research indicates that physical exercise stimulates neurogenesis in the brain, while cognitive training promotes synaptic formation. Combined, these interventions yield greater cognitive benefits than either approach alone [130,132]. By simultaneously developing physical and cognitive abilities, these integrated interventions strengthen the connection between the mind and body, facilitating faster and more efficient neuromuscular responses. For instance, the inherent rhythmic movements in Tai Chi improve muscle control and mental agility, leading to quicker reaction times and enhanced balance [133,134].

Dual-task training, which incorporates cognitive challenges into physical activities, enhances gait stability and cognitive function, establishing its importance in fall prevention programs [135]. Furthermore, aerobic activities like brisk walking and cycling contribute to cognitive health by improving cardiovascular function and cerebral blood flow, ensuring a consistent supply of oxygen and nutrients to the brain. These physiological benefits support cognitive functions related to decision-making and attentiveness, which are vital for the safe navigation of complex environments [136,137].

By integrating physical and cognitive training, multidisciplinary approaches offer a robust framework for improving stability, mobility, and cognitive resilience, ultimately reducing the risk of falls in older adults.

### 6.2. Holistic Intervention Models

Holistic intervention models offer a multidimensional approach to fall prevention by integrating physical, cognitive, and social components into comprehensive programs [138]. Programs such as the Otago Exercise Program (OEP) and Stepping On exemplify these models, combining physical training with cognitive exercises and social engagement to address the multifaceted nature of fall risk. Developed in New Zealand, the OEP incorporates warm-up activities, strength training, balance exercises, and walking practices. Evidence demonstrates that the OEP enhances cognitive function, lower limb strength, balance, and postural control in older adults, effectively reducing fall risk. Additionally, it has been associated with improved well-being and reduced symptoms of depression in older populations [139]. These programs emphasize physical improvements and strategies for navigating fall risks in daily life [13,138,139,140,141].

The layered and interdependent structure of holistic models is particularly impactful. The physical components focus on improving muscle strength, joint stability, and dynamic balance, directly addressing physical fall risk factors. Cognitive exercises, such as memory and decision-making tasks, effectively enhance participants’ ability to respond to potential fall risks [138,139]. The social component, including group exercise sessions, creates a supportive and interactive environment that fosters emotional well-being and improves adherence to the program. This social interaction promotes a sense of accountability and helps alleviate psychological barriers, such as the fear of falling [140,141].

The research conducted underscores the effectiveness of these multidimensional approaches. Participants in holistic intervention programs report significantly fewer falls than single-component program participants. This finding highlights the importance of addressing multiple dimensions of well-being—physical, cognitive, and social—in fall prevention strategies [142,143]. By offering a comprehensive and interconnected framework, holistic intervention models provide a sustainable and effective means of reducing fall risk and enhancing the overall quality of life for older adults.

### 6.3. Technological Supports and Feedback Mechanisms

Technological advancements have significantly enhanced the effectiveness and safety of exercise-based fall prevention programs. Wearable sensors, such as accelerometers and gyroscopes, enable the real-time monitoring of movement patterns, balance, and stability. These devices provide immediate feedback, allowing for the customization of exercise interventions to suit individual needs. This personalization facilitates on-the-spot adjustments to posture or gait during exercise, reinforcing correct movement patterns and reducing fall risk [144,145,146,147].

Feedback tools like force plates and pressure-sensitive insoles deliver detailed data on weight distribution and movement symmetry, which are crucial for assessing balance and stability. Healthcare providers can use these data to design targeted interventions that address deficiencies, improve overall balance, and reduce fall risk. For example, gait analysis revealing asymmetries can lead to tailored unilateral exercises to correct these imbalances [148].

Virtual reality (VR) and exergaming platforms have transformed fall prevention strategies by offering engaging, simulated environments that challenge balance and cognitive functions. These immersive tools allow older adults to safely practice navigating complex scenarios, enhancing their ability to respond to real-world obstacles. Data collected from these interactive sessions support continuous program adjustments, enabling personalized progress and fostering adherence [144,149].

Integrating technological tools into exercise programs makes them more adaptive, personalized, and engaging, encouraging sustained participation. The combination of real-time feedback and adaptive technologies bridges the gap between physical and cognitive improvements, reinforcing both components and maximizing the potential for effective fall prevention [145,149]. For a detailed analysis of integrated mechanisms in multidisciplinary approaches, see Table 4 in this study.

## 7. Future Directions and Research Needs

Advancements in exercise science provide significant opportunities to improve fall prevention strategies, explicitly enhancing balance, stability, and cognitive engagement for older adults [150]. Given the multifactorial nature of falls, future research should adopt a cross-disciplinary approach involving physiologists, neurologists, psychologists, biomechanists, and geriatricians to better understand how muscle strength, neuromuscular coordination, and cognitive processes collectively influence balance and stability [151].

### 7.1. Development and Evaluation of Dual-Task Training Programs

Future research should prioritize the efficacy of dual-task training programs, combining physical and cognitive exercises to enhance motor and cognitive functions [152] simultaneously. For example, integrating balance exercises with tasks requiring memory recall or problem-solving could improve the ability of older adults to manage complex real-world situations. Large-scale randomized controlled trials (RCTs) are needed to establish standardized protocols and assess their effectiveness across diverse populations.

### 7.2. Long-Term Studies on Intervention Outcomes

Long-term, longitudinal studies are crucial to evaluating the sustained benefits of regular balance and strength training on muscle mass, neuromuscular function, and cognitive resilience [153]. These studies should explore whether early interventions lead to lasting reductions in fall risk and foster independence in older adults. Such research will provide essential data for updating public health guidelines and exercise recommendations.

### 7.3. Implementation of Multi-Component Programs

Research should focus on developing and evaluating multi-component exercise programs that integrate strength, balance, flexibility, and cognitive training [154]. Comparative studies between multi-component and single-component interventions can provide valuable insights into their relative efficacy. Programs should include practical, scalable models for implementation in both community and clinical settings, such as those combining group sessions with individualized home exercises.

### 7.4. Exploration of Exercise-Nutrition Synergy

Future investigations should assess the interaction between exercise and nutritional support, including protein supplementation and vitamin D intake, in mitigating sarcopenia and enhancing bone density. Additionally, research into pharmacological treatments that complement exercise interventions could offer a more holistic approach to fall prevention. These studies should aim to develop integrated guidelines that optimize exercise and nutrition for older adults.

### 7.5. Cost-Effectiveness and Resource Allocation

Given the economic burden of falls, cost-effectiveness analysis is essential to inform resource allocation and public health policy [155]. Research should compare the costs and benefits of various interventions, such as in-person group classes, home-based resistance training, and technology-assisted programs (e.g., virtual reality platforms). Understanding the potential cost savings from reduced hospitalizations and long-term care needs can guide the adoption of impactful fall prevention practices.

### 7.6. Advancement of Technology-Driven Interventions

Emerging technologies like wearable sensors, mobile health applications, and virtual reality should be integrated into fall prevention research. Wearable devices that monitor balance, mobility, and stability in real-time can provide personalized feedback. At the same time, virtual reality platforms can simulate real-world scenarios to enhance neuromuscular coordination and cognitive engagement. Studies should assess such technology-driven interventions’ feasibility, scalability, and long-term benefits.

### 7.7. Healthcare Provider Training and Collaboration

Future initiatives should include developing comprehensive training programs for healthcare providers, focusing on fall risk assessment and evidence-based intervention strategies. Collaboration between physiotherapists, occupational therapists, fitness trainers, and medical professionals will enable the creation of coordinated care plans tailored to the individual needs of older adults [154]. Regular interdisciplinary communication can ensure continuous improvement in intervention effectiveness.

### 7.8. Personalized Intervention Strategies

Tailoring interventions to individual health profiles, including physical capabilities, medical history, and psychological factors, will improve adherence and optimize outcomes. Research should explore strategies for personalizing exercise regimens, incorporating adaptive technologies, and delivering interventions in varied settings to address the unique needs of older adults.

By addressing these practical and research-oriented proposals, future efforts can advance evidence-based practices, improve health outcomes, and enhance the sustainability of fall prevention initiatives for older adults. Such initiatives will promote active aging, reduce fall-related healthcare costs, and improve the overall quality of life for older adults.

## 8. Conclusions

The conclusion of this integrative review underscores the essential role exercise interventions play in reducing fall risk among older adults through biomechanical, physiological, and psychological mechanisms. Critical elements of effective fall prevention programs include exercises that enhance balance, gait stability, and postural control, thereby strengthening lower body muscles, improving joint mobility, and refining response times. Physiologically, resistance and weight-bearing activities counter age-related declines in muscle mass and bone density while enhancing neuromuscular coordination for rapid responses to balance disruptions. Psychologically, targeted exercise programs alleviate fear of falling, build confidence, and boost cognitive functions like attention and spatial awareness, supporting safer mobility.

This review highlights the most effective mechanism-driven, multidimensional exercise programs that combine strength training, balance improvement, cognitive challenges, and confidence building. To maximize their impact, healthcare professionals and policymakers should prioritize such evidence-based strategies to promote active aging and enhance the quality of life for older adults. Future research should focus on cross-disciplinary studies to explore the interplay between these mechanisms and on long-term evaluations to determine lasting benefits, ultimately contributing to the sustainability of fall prevention initiatives.

## Figures and Tables

**Table 1:** Summary of biomechanical mechanisms of fall prevention through exercise.

Main Category	Sub-Category	Detailed Summary	Citations
Balance and Postural Control		Balance and postural control are enhanced through targeted exercises that improve stability and proprioception. Exercises include single-leg stances, balance board activities, and core training (e.g., Pilates). These activities activate proprioceptors in muscles, tendons, and joints, which sharpen the body’s responses to balance disturbances, improving fall resilience.	[13,14,15,16,17,18,19,20]
	Proprioception Enhancement	Regular physical activities preserve proprioception by improving the sensitivity of muscle spindles and other proprioceptive sources, facilitating more accurate postural adjustments. Enhanced proprioception ensures faster reflexive responses and overall improved balance.	[13,14,15,16]
	Neuromuscular Coordination	Balance exercises require coordinated muscle activation supported by neuromuscular systems involving the brain, spinal cord, and neural networks. Training that requires trunk muscle strength and core stability significantly improves neuromuscular communication and balance performance.	[17,18,19,20]
Gait and Lower Limb Strength		Gait stability and lower limb strength are vital for stable movement. Age-related sarcopenia impacts muscle mass and strength, affecting gait mechanics and balance. Resistance training, including squats, leg presses, and lunges, improves muscle hypertrophy, joint support, and functional capacity, aiding in safer movements.	[27,28,29,30,31,32,33,34,35,36,37,38]
	Gait Mechanics	Proper gait depends on coordinated muscle function, joint mobility, and neuromuscular control. Exercises targeting lower limb strength help maintain standard gait patterns, reducing fall risks by supporting joint function and enhancing shock absorption.	[29,30,31,32,33,34,35]
	Resistance Training Benefits	Programs involving resistance exercises promote muscle hypertrophy, improve gait speed, and enhance neuromuscular activation. Recommendations often include 1–3 sets of 6–15 repetitions at 30–70% of 1-RM for optimal results.	[32,33]
Joint Stability and Range of Motion		Flexibility and joint stability are improved through dynamic and static stretching. Regular flexibility exercises (e.g., hip circles and ankle dorsiflexion) reduce muscle stiffness, enhance joint mobility, and promote shock absorption. These adaptations are essential for safer movement and fall prevention.	[45,46,47,48,49,50,51,52,53,54]
	Targeted Joint Exercises	Hip and ankle mobility drills enhance joint stability, enabling smoother movement transitions. Enhanced flexibility supports better load distribution and impact absorption, reducing injury risk during falls.	[48,49,50,51]
	Proprioceptive Feedback	Improving joint range of motion through stretching enhances mechanoreceptor sensitivity, providing superior proprioceptive feedback that aids postural control.	[47,52,53,54]
Dynamic Stability		Dynamic stability involves maintaining balance during movement, using sensory inputs, neuromuscular responses, and musculoskeletal coordination. Step-up routines, agility drills, and lateral movements improve reaction time and proprioception. These exercises facilitate quick adaptations to sudden changes in balance, which is crucial for fall prevention.	[54,55,56,57,58,59,60,61,62,63,64]
	Agility and Reflex Training	Agility drills (e.g., cone weaves and shuttle runs) train the body for lateral stability and quick reactions. Such training helps prevent falls due to sideward balance loss.	[59,60,61,62]
	Multisensory Integration	Balance training using unstable surfaces engages the vestibular and proprioceptive systems, teaching the brain to integrate sensory information for more effective balance control in dynamic conditions.	[54,63,64]

**Table 2:** Summary of physiological mechanisms underlying fall prevention.

Main Category	Sub-Category	Detailed Summary	Citations
Muscle Hypertrophy and Sarcopenia Mitigation		Age-related sarcopenia, characterized by a decline in muscle mass and strength, significantly increases fall risk. Resistance training counteracts sarcopenia by promoting muscle hypertrophy, enhancing muscle cross-sectional area and strength, and improving functional capacity. Squats and leg presses stimulate muscle protein synthesis and fiber recruitment.	[28,30,31,32,37,65,66,67,68,69,70]
	Muscle Fiber Activation	Resistance training targets type II muscle fibers, which are crucial for rapid and forceful movements. Enhanced activation and hypertrophy of these fibers improve balance recovery and reduce fall risks. Regular training stimulates anabolic hormones and neuromuscular adaptations for better strength and coordination.	[30,37,67,68]
Neuromuscular Adaptations		Neuromuscular coordination is vital for stability and fall prevention. Strength and balance training improve motor unit recruitment and synchronization, leading to quicker and more efficient muscle responses. Enhanced motor unit discharge rates and reduced recruitment-threshold forces contribute to better muscle reaction and balance control.	[36,71,72,73,74,75,76,77,78,79]
	Motor Unit Recruitment	Strength training enhances the efficiency of motor units by improving their recruitment and firing rates. This results in increased force generation to maintain balance during sudden movements.	[72,73,74]
	Proprioceptive and Nervous System Response	Balance exercises enhance neuromuscular control, improving proprioceptive accuracy and response to perturbations. Enhanced proprioception supports better body position detection and postural adjustments, which are essential for preventing falls.	[75,76,77,78]
Bone Density and Joint Integrity		Osteopenia and osteoporosis are common in older adults and increase fracture risk from falls. Weight-bearing and resistance exercises stimulate bone remodeling by applying stress to bones, enhancing osteoblast activity, and promoting mineral deposition. Improved joint integrity from more robust muscles and tendons improves joint stability and alignment.	[80,81,82,83,84,85,86]
	Bone Remodeling	Mechanical stress from weight-bearing activities encourages bone density maintenance and increases mineral content, thereby reinforcing bone strength and reducing the risk of fractures during falls.	[82,83,84,85]
	Joint Stability Support	Strengthened muscles and tendons surrounding joints alleviate joint stress and support proper alignment, reducing fall risk and injury severity.	[86]
Cardiovascular and Endurance Benefits		Improved cardiovascular endurance supports prolonged physical activity without undue fatigue, which is essential for balance and fall prevention. Aerobic exercises enhance cardiac output, lung capacity, and oxygen utilization, leading to more incredible stamina and reduced fatigue. These adaptations contribute to sustained physical performance and better postural control.	[87,88,89,90,91]
	Aerobic Training Effects	Activities like brisk walking and cycling improve cardiovascular health and promote efficient oxygen delivery to muscles, enhancing endurance and reducing the risk of exhaustion-related falls.	[88,89,90]
	Fatigue Reduction	Improved endurance delays the onset of fatigue, allowing older adults to maintain stability and quick reaction times during physical activities.	[85,86,87,88,89,90,91]

**Table 3:** Summary of psychological mechanisms in exercise-based fall prevention.

Main Category	Sub-Category	Detailed Summary	Citations
Fear Reduction and Confidence Building		The fear of falling is a significant barrier to activity in older adults, leading to reduced mobility and increased fall risk. Exercise programs incorporating gradual exposure, positive reinforcement, and goal setting help build confidence and reduce fear. Improved body control through balance and strength training reinforces self-efficacy and motivates continued participation in physical activities.	[92,93,94,95,96,97,98,99,100]
	Psychological Barriers	Fear and anxiety can impair physical performance by altering gait and increasing muscle tension. Addressing these through targeted exercise can restore confidence and reduce psychological fall risk factors.	[93,94]
	Social Support Influence	Group-based exercise settings enhance confidence through social interaction and mutual encouragement, reducing feelings of isolation and promoting adherence. Supportive environments facilitate psychological resilience and sustained activity.	[99,100]
Cognitive Enhancement		Exercise positively influences cognitive function, which is essential for safe navigation and decision-making. Activities like Tai Chi, dance, and aerobic exercises enhance attention, executive function, and spatial awareness. These cognitive improvements translate to better reaction times and environmental awareness, reducing fall risk.	[97,101,102,103,104,105,106,107,108,109]
	Dual-Task Performance	Combining cognitive tasks with physical exercises (e.g., dual-task training) strengthens the ability to maintain balance while multitasking, a common requirement in daily life. This approach supports overall cognitive agility and enhances safe mobility.	[107,108,109]
	Brain Health and Neurogenesis	Physical activities increase cerebral blood flow and stimulate the release of neurotrophic factors like BDNF, supporting neuron health and cognitive function. This promotes mental sharpness, aiding in faster and more accurate decision-making necessary for fall prevention.	[97,105,106,107,108,109]
Mood and Motivation		Regular physical activity releases endorphins and neurotransmitters such as serotonin and dopamine, improving mood and reducing stress. Enhanced mood contributes to better engagement in exercise routines, which is crucial for long-term fall prevention strategies.	[110,111,112,113,114,115,116,117,118,119]
	Reduced Depression and Anxiety	Exercise mitigates symptoms of depression and anxiety, which are common in older adults and negatively impact motivation and physical activity. Addressing these symptoms through exercise encourages sustained participation and active lifestyles.	[104,105]
	Social Interaction Benefits	Group exercises provide opportunities for social engagement, which improves mood and boosts motivation through collective support and encouragement. Social activities within exercise programs help combat loneliness, fostering adherence and improving mental well-being.	[114,115,116]
Social Influence and Support		Social engagement in exercise programs reduces isolation and fosters a sense of community. Group-based activities create accountability and motivate participants through shared goals and peer encouragement. Instructors play a vital role in creating inclusive environments, enhancing participation, and building psychological resilience.	[44,120,121,122,123,124,125,126,127]
	Emotional and Practical Support	Group settings offer emotional reinforcement and practical assistance, increasing adherence to exercise programs. This social structure bolsters emotional well-being and promotes sustained physical activity, reducing fall risk.	[123,124,125,126,127]

**Table 4:** Summary of integrated mechanisms in multidisciplinary approaches.

Main Category	Sub-Category	Detailed Summary	Citations
Holistic Exercise Programs		Multidisciplinary exercise programs combine strength, balance, flexibility, and cognitive training to address multiple aspects of fall prevention. This integrated approach leverages the benefits of each type of exercise to enhance overall physical and mental capabilities, providing a comprehensive solution for reducing fall risk in older adults.	[128,129,130,131,132,133,134,135]
	Combined Modalities	Programs incorporating Tai Chi, Pilates, strength training, and cognitive exercises promote synergy between physical and mental health, optimizing muscle function and cognitive performance for better balance and fall prevention.	[130,131,132,133]
	Personalized Training Plans	Tailored exercise regimens considering individual health conditions, preferences, and physical capabilities result in better adherence and effectiveness. Personalized plans improve participation and maximize the benefits of multidisciplinary approaches.	[134,135]
Collaboration Between Healthcare Professionals		Effective fall prevention strategies often involve collaboration between physiotherapists, occupational therapists, fitness trainers, and medical professionals. This team-based approach ensures a comprehensive assessment of an individual’s risk factors and the development of targeted interventions that include exercise, lifestyle modifications, and medical guidance.	[136,137,138,139,140,141,142]
	Coordinated Care Plans	Multi-disciplinary care plans integrate various healthcare professionals’ expertise to enhance interventions’ effectiveness by addressing all aspects of a person’s physical and mental health. This coordination supports seamless care transitions and maximizes patient safety.	[139,140,141,142]
	Interdisciplinary Communication	Regular communication between different healthcare providers ensures the continuity of care and consistent progress monitoring, making adjustments to maintain or improve the patient’s fall prevention strategy.	[137,138,139,140]
Technological Integration in Exercise		Incorporating technology, such as wearable devices and virtual reality (VR), enhances the effectiveness of multidisciplinary fall prevention programs. These tools provide real-time feedback, monitor progress, and facilitate interactive exercises that improve engagement and adherence.	[143,144,145,146,147,148,149]
	Wearable Technology	Devices that track movement, balance, and heart rate help healthcare providers monitor exercise effectiveness and identify potential fall risks early. This data-driven approach enhances personalized care and feedback for participants.	[144,145,146,147]
	Virtual Reality and Simulations	VR-based exercises simulate real-life scenarios to challenge balance and cognitive function safely. Such simulations improve situational awareness and adaptability, which are crucial for preventing falls in natural environments.	[148,149]

## Data Availability

Data are contained within the article.
